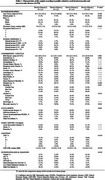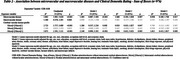# Vascular disease profiles and their association with cognitive abilities: a clinical pathological study

**DOI:** 10.1002/alz70860_106461

**Published:** 2025-12-23

**Authors:** Raul dos Reis Ururahy, Alberto Fernando Oliveira Justo, Renata Elaine Paraizo Leite, Vitor Ribeiro Paes, Roberta Rodriguez, Lea T. Grinberg, Carlos Augusto Pasqualucci, Eduardo Ferriolli, Claudia Kimie Suemoto

**Affiliations:** ^1^ Biobank for Aging Studies of the University of São Paulo, São Paulo, São Paulo, Brazil; ^2^ Memory and Aging Center, UCSF Weill Institute for Neurosciences, University of California, San Francisco, San Francisco, CA, USA; ^3^ University of Sao Paulo Medical School, São Paulo, Brazil

## Abstract

**Background:**

Although the association between brain infarcts and cognitive impairment has been widely investigated, the contribution of vascular pathology to cognitive performance has been less studied. In this population‐based study, we sought to better understand the clinical and epidemiological characteristics of vascular pathology phenotypes and evaluate their association with cognitive abilities.

**Method:**

Sociodemographic/clinical data and cognitive abilities were assessed with an informant. Hyaline arteriolosclerosis (HA) was evaluated in 13 brain areas, and categorized as absent, mild, moderate, or severe. Participants with moderate/severe HA were considered positive for microvascular disease (Micro). Intracranial atherosclerosis was assessed on Circle of Willis' arteries, and classified as mild, moderate, or severe. Cases with moderate/severe obstructions were considered positive for macrovascular disease (Macro). Cross‐sectional data was used to compare the four vascular pathology profiles based on microvascular and macrovascular disease status (Micro[+]/Macro[+]; Micro[+]/Macro[‐]; Micro[‐]/Macro[+]; and Micro[‐]/Macro[‐]). Linear regression models adjusted for sociodemographic and clinical variables, as well as for the presence of brain macro and microinfarcts, were used to investigate the association of cognition with the four vascular pathology profiles. We also investigated the associations of cognition with micro and macrovascular pathologies alone.

**Result:**

In 976 participants, the mean age was 75.4±13.0 years old, 47.9% were women, and 60.5% were white. Microvascular disease was more frequent (38.1%) than macrovascular (27.1%). Regarding vascular pathology profiles, 26.1% of participants were Micro[+]/Macro[‐], 15.1% were Micro[‐]/Macro[+], and 12.0% were Micro[+]/Macro[+]. Participants in the Micro[+] groups were older, more frequently women, had lower body mass index, and worse cognitive abilities. Vascular groups were similar regarding the distribution for most clinical comorbidities. Furthermore, Micro[+] groups had more frequent neuropathological diagnoses of Alzheimer's disease and vascular dementia. An association was observed between Microvascular group and worse cognitive abilities, even after adjustment for a broad set of variables. No association with cognition was observed for Macrovascular group. When we considered the 4 vascular disease profiles, only the Micro[+]/Macro[+] group was associated with worse cognitive measures.

**Conclusion:**

Microvascular disease alone or in combination with macrovascular disease was associated with worse cognitive abilities.